# Digital campus governance and cultural identity of multi-ethnic students: a multiple mediation analysis of online social support and sense of community connection

**DOI:** 10.3389/fpsyg.2026.1918341

**Published:** 2026-08-20

**Authors:** Xiamu Yongzhong

**Affiliations:** College of Literature, Sichuan Minzu College, Kangding, China

**Keywords:** campus sense of community connection, digital campus governance, ethnic-cultural identity, moderated chain mediation, multi-ethnic students, online social support

## Abstract

**Introduction:**

Drawing upon Socio-Technical Systems Theory and the Sense of Community Theory, this study examines the associations between digital campus governance participation and the ethnic-cultural identity of multi-ethnic college students, testing the multiple indirect associations of online social support and campus sense of community connection, as well as the potential moderating role of ethnic background.

**Methods:**

Utilizing a cross-sectional questionnaire survey method, data were collected from 890 students across multi-ethnic universities in Southwest China.

**Results:**

Regression and bias-corrected Bootstrap analyses indicate that: (1) digital campus governance participation is significantly and positively associated with ethnic-cultural identity; (2) online social support and campus sense of community connection exhibit significant parallel indirect statistical covariations in this relationship, while jointly constructing a sequential chain association structure defined as “digital participation → online social support → campus sense of community connection → ethnic-cultural identity”; (3) the moderating role of ethnic background on the relationship between digital participation and online social support is not statistically significant. This suggests that the positive association of digital participation possesses cross-group similarity and stability between Han and ethnic minority students.

**Discussion:**

This study provides micro-psychological empirical evidence for the construction of inclusive digital campuses.

## 1 Introduction

Against the backdrop of the continuous deepening of social digital transformation, the organizational boundaries, interaction patterns, and governance logic of contemporary Chinese universities are being reshaped. University campuses are no longer merely physical spaces composed of classrooms, dormitories, cafeterias, and administrative buildings, but are gradually becoming composite living fields woven together by WeChat groups, campus service platforms, online feedback systems, campus forums, and various digital communities. For today’s college students, understanding campus life requires not only observing their offline interactions in classrooms, clubs, or dormitories but also entering those digital spaces constituted by data, platforms, notifications, comments, and feedback.

Particularly in university environments where students from diverse ethnic backgrounds live together, the significance of digital campus governance is even more complex. It concerns not only administrative efficiency, information transparency, and convenience in handling transactions, but may also form subtle statistical associations with students’ support experiences, perceptions of belonging, and ethnic-cultural identities. It is against this background that this study attempts to systematically test the relationships among digital campus governance participation, online social support, campus sense of community connection, and ethnic-cultural identity.

### 1.1 Research background and origin of the problem

With the gradual popularization of digital governance infrastructure represented by WeChat groups, campus-specific administrative Apps, online service halls, and campus BBS, university management models no longer remain at the stage of one-way administrative notifications and offline transaction processing. Instead, they are gradually entering a highly interconnected stage of a “digital campus community.” Through these platforms, students obtain notifications, submit requests, participate in voting, and provide feedback. They also conduct mutual information assistance, emotional expression, and relationship maintenance in semi-formal or informal network spaces.

In China’s southwestern region, where universities have a relatively high concentration of multi-ethnic students, the campus itself is a typical field of multicultural interaction. Students from different ethnic groups, regions, language habits, and life backgrounds enter the same campus system and digital governance system. For them, campus digital platforms are not only tools for administrative affairs but may also serve as important windows to observe whether the school responds to their needs, whether peers are willing to provide help, and whether they are accepted by the community.

Existing studies usually discuss digital campus governance from the perspectives of technical efficiency or educational management. However, they rarely ask further: Is students’ participation level in digital campus governance platforms systematically associated with their perceived online social support, campus sense of community connection, and ethnic-cultural identity? If an association exists, is it merely a simple co-directional change of variables, or does it form a more complex indirect path through several psychological mediating variables? Does this association model differ significantly between Han and ethnic minority students?

Based on the above awareness of these issues, this study takes digital campus governance participation as the core explanatory variable, online social support and campus sense of community connection as mediating variables, and ethnic-cultural identity as the outcome variable in an attempt to establish a multiple mediation model. It must be emphasized that because this study adopts a cross-sectional questionnaire design, all path interpretations throughout the text are strictly limited to the level of statistical association and do not make any causal assertions, nor can they exclude the possibility of reverse covariation in real-world contexts.

### 1.2 Theoretical perspective and research significance

This study mainly constructs its analytical framework using Socio-Technical Systems Theory, the Sense of Community Theory, the Common Ingroup Identity Model (CIIM) (Gaertner and Dovidio, 2014), and Bicultural Identity Integration Theory (BIIT).

Socio-Technical Systems Theory emphasizes that technical systems do not operate in isolation but are always embedded in specific organizational structures, social relations, and psychological experiences. On the surface, university digital governance platforms are tools for information transmission and transaction processing, but their actual operation is linked to students’ sense of participation, being responded to, trust, and support. In other words, digital campus governance is not a pure technical arrangement, but a composite field under the joint action of technical systems and campus social systems.

The Sense of Community Theory (McMillan and Chavis, 1986) points out that an individual’s experience of belonging to a certain community is usually related to their sense of membership, influence, integration and fulfillment of needs, and shared emotional connection. For young students who leave their original families and local cultural environments to enter university, the campus sense of community connection is not naturally formed. Instead, it is gradually accumulated through continuous interaction, institutional responsiveness, and emotional confirmation. Although digital platforms exist online, they may be linked to students’ perceptions of the physical campus community. As Blanchard and Markus (2004) pointed out, with the normalization of online interaction, shared experiences in virtual spaces can not only trigger a virtual sense of community but also further project and transform into identity with the physical community due to members’ stickiness to the digital ecology.

**Figure 1:**
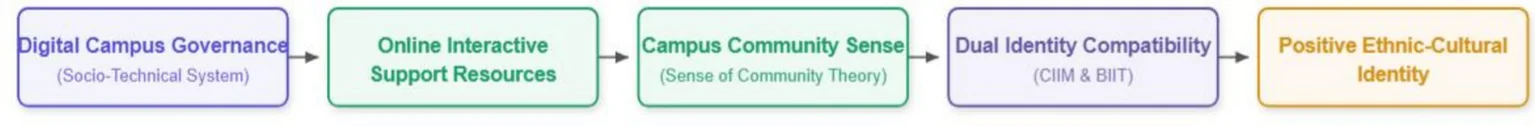
The integrated theoretical logic chain of the study.

Combining the two theories, these theoretical lenses, this study constructs an integrated analytical framework (see the theoretical logic diagram structurally mapped in Figure 1 for reference). Digital campus governance participation (operating as a socio-technical system) may be related to online interactive support experiences, which in turn may be associated with students’ sense of belonging to the physical campus community (Sense of Community Theory). Furthermore, according to CIIM and BIIT, acquiring an overarching inclusive identity (such as campus belonging) does not necessarily dilute individuals’ original ethnic identity. Instead, an accepted and supportive campus community provides the psychological safety and compatibility necessary for multi-ethnic students to maintain dual identities, which statistically correlates with their positive evaluation of their own ethnic-cultural background. Therefore, this study not only helps extend the psychological explanation dimension of digital governance research but also provides empirical evidence for multi-ethnic universities to build a more inclusive digital campus environment.

## 2 Literature review and research hypotheses

This study focuses on four core constructs: Digital Campus Governance Participation (also referred to as Digital Community Participation, DCP), Online Social Support (OSS), Sense of Community Connection (SoCC), and Ethnic-Cultural Identity (EI). Among them, ethnic-cultural identity mainly refers to students’ subjective sense of identification with their own ethnic background, cultural traditions, and group belonging. Its connotation is close to the ethnic identity proposed by Phinney and Ong, rather than a generalized cultural identity.

### 2.1 Digital campus governance participation and ethnic-cultural identity

The relationship between digital technology and individual identity has always been an important topic in cyberpsychology, sociology of education, and communication studies. Early views experienced the anxiety of the “Internet paradox,” worrying that the de-socializing characteristics of online interaction would deprive people of their physical offline involvement and weaken individuals’ attachment to local, ethnic, and traditional cultures (Kraut et al., 1998). However, as digital platforms have gradually extended from entertainment spaces to important media for public participation, organizational governance, and social support, more and more studies have begun to notice that digital participation does not necessarily mean the dissolution of cultural identity. Instead, an ongoing academic debate exists: some argue that standardized digital network platforms may promote cultural homogenization and dilute minority identities (Croucher, 2011), while others counter that digital spaces offer marginalized groups safe, low-barrier enclaves for distinct cultural expression and network building (Tynes et al., 2012). This study aims to provide precise marginal contributions to this dispute within the context of multi-ethnic higher education.

In the university context, digital campus governance participation includes not only browsing notifications and completing online transactions but also returning feedback through platforms, participating in public issue discussions, and engaging in mutual assistance and expression in campus communities. For multi-ethnic students, these digital participation experiences may be related to their understanding of their own identity positions. Especially during the key transition period of self-identity exploration when young students enter universities (Branje et al., 2021), a campus digital space that can be seen, responded to, and allow students to express their needs may be positively associated with students’ more positive ethnic-cultural identity levels.

Accordingly, the following hypothesis is proposed:

*H1*: The level of digital campus governance participation is significantly and positively associated with ethnic-cultural identity.

### 2.2 The mediating association of online social support

Online social support refers to the emotional, informational, and companionship support perceived by individuals in online environments. In the context of higher education, digital platforms often constitute important support systems for students to accumulate social capital, buffer academic adaptation and interpersonal pressure (Dost and Smith, 2023), and establish horizontal emotional connections (Ellison et al., 2007). In particular, when students face academic pressure, interpersonal adaptation, life difficulties, or cultural differences, online spaces may provide low-barrier channels for seeking help and receiving responses.

Students with higher levels of digital campus governance participation usually have more opportunities to access campus online communities and public platforms, and are also more likely to obtain information, feedback, and emotional responses in these spaces. Existing research on college students shows that online social support plays an important mediating role between Internet use and student psychological adaptation (Luo et al., 2022). This provides an empirical basis for placing OSS in the indirect association path between DCP and EI in this study. This is consistent with the results of recent domestic empirical studies on Chinese university students, which show that active online interaction and communication behavior can statistically covary with individuals’ perceived online social support (Jiang et al., 2023; Xin et al., 2024). At the same time, as an important psychological resource, online social support may also be related to students’ ethnic-cultural identity levels. For multi-ethnic students, supported and understood online experiences may be statistically linked to their more positive view of their own cultural background.

Accordingly, the following hypothesis is proposed:

*H2*: Online social support forms a significant mediating association between digital campus governance participation and ethnic-cultural identity.

### 2.3 The mediating association of campus sense of community connection

Campus sense of community connection reflects students’ sense of membership, emotional attachment, and involvement in their university community. Compared with online social support, which emphasizes help and response in specific interactions, campus sense of community connection is closer to an overall, stable experience of community connection.

Digital campus governance participation may be associated with campus sense of community connection. On the one hand, through participating in campus affairs, obtaining information, and expressing demands on online platforms, students may more easily feel that they are members of the campus community. On the other hand, the feedback efficiency and interactive atmosphere of campus platforms may also be related to students’ sense of belonging to the overall school environment. Relevant studies have also shown that the experience of using campus digital technology facilities may affect college students’ sense of school identity and belonging, indicating that digital governance is not a simple technical management process but may also enter students’ psychological evaluation of the campus community (Wang et al., 2022).

Ethnic-cultural identity does not necessarily conflict with campus belonging. Drawing upon the Common Ingroup Identity Model (CIIM) (Gaertner and Dovidio, 2014) and Bicultural Identity Integration Theory (BIIT), forming a superordinate identity (such as university community membership) does not require the abandonment of distinctive subgroup identities (such as ethnic background), provided the institutional environment is perceived as culturally inclusive. For multi-ethnic students, acceptance by the campus community may provide psychological safety for expressing and understanding their own ethnic cultural identity. In other words, when students feel respected and accepted in school, their positive evaluation of their own ethnic-cultural background may also be more fully presented and integrated harmoniously without psychological tension.

Accordingly, the following hypothesis is proposed:

*H3*: Campus sense of community connection forms a significant mediating association between digital campus governance participation and ethnic-cultural identity.

### 2.4 The chain mediating association of online social support and campus sense of community connection

Online social support and campus sense of community connection are not isolated from each other. Students’ experiences of obtaining help, responses, and companionship on digital platforms may be linked to their overall sense of belonging to the physical campus community. This association of online support extending to physical cohesion has also been partially confirmed in chain mediation studies of Chinese university student groups, reflecting that the support captured online by individuals can be transformed into an overall connection to the group by enhancing their psychological capital (Qian et al., 2023).

In the model of this study, digital campus governance participation may first be related to online social support, which is further related to campus sense of community connection, and campus sense of community connection is in turn linked to ethnic-cultural identity. The rationale for this assumed sequential structure aligns with Media Dependency Theory (Ball-Rokeach and DeFleur, 1976), which views initial digital interactions as accessible initial gateways for capturing immediate support (OSS), a process that subsequently precipitates into a more stable institutional belonging (SoCC) and positive identity (EI). However, it must be acknowledged that cross-sectional data inherently limits causal inferences, and multiple alternative reverse association pathways cannot be strictly ruled out. For instance, students with a highly consolidated ethnic-cultural identity (EI) might naturally be more motivated to seek out and integrate into campus public spaces (SoCC), and those with strong pre-existing community ties are inherently more likely to actively utilize digital governance platforms (DCP). Therefore, the proposed pathway does not imply a strict temporal causal order but represents a chain indirect association structure that fits theoretical expectations in cross-sectional data.

Accordingly, the following hypothesis is proposed:

*H4*: Online social support and campus sense of community connection form a significant chain mediating association between digital campus governance participation and ethnic-cultural identity, i.e., DCP → OSS → SoCC → EI.

### 2.5 The potential moderating association of ethnic background

In multi-ethnic university settings, students from different ethnic backgrounds may have different language experiences, cultural expression habits, offline communication networks, and campus adaptation needs. Ethnic identity is also affected by the school interaction environment and institutional response methods; studies based on Chinese school contexts have found that students’ perception of teachers’ ethnic-racial socialization practices is significantly associated with their ethnic identity and mental health (Lai et al., 2024). Therefore, whether the digital campus platform can provide a culturally sensitive and responsive interactive environment may relate to the perception of online support and campus acceptance among students of different ethnic backgrounds. Some studies suggest that digital spaces can provide complementary channels of expression for ethnic minority students, allowing them to obtain information and support in low-pressure environments; as shown in the intersectional study of marginalized and minority groups by Gordon et al. (2023), minority groups and mainstream groups have systematic interactions in their support-seeking strategies and perceptual sensitivity on social media, where marginalized identities often lead to more obvious reliance on online support. Therefore, this study further examines whether ethnic background plays a moderating role in this process. If ethnic minority students rely more on online platforms for information and support, the association between DCP and OSS may present stronger statistical features in the ethnic minority student group. However, this judgment still needs to be confirmed through statistical testing.

Accordingly, the following hypothesis is proposed:

*H5*: Ethnic background moderates the association characteristics between digital campus governance participation and online social support, and the strength of this association differs significantly between Han students and ethnic minority students.

Based on the above theoretical derivation, this study constructs an integrated multiple mediation association model that simultaneously contains direct associations, parallel dual mediation, chain mediation, and a moderated first stage. This model is dedicated to systematically answering: how does digital campus governance participation generate spillover effects on the cultural identity of multi-ethnic college students through dual online and physical psychological resources (social support and community connection), and whether this association feature remains stable across groups with different ethnic backgrounds.

**Figure 2:**
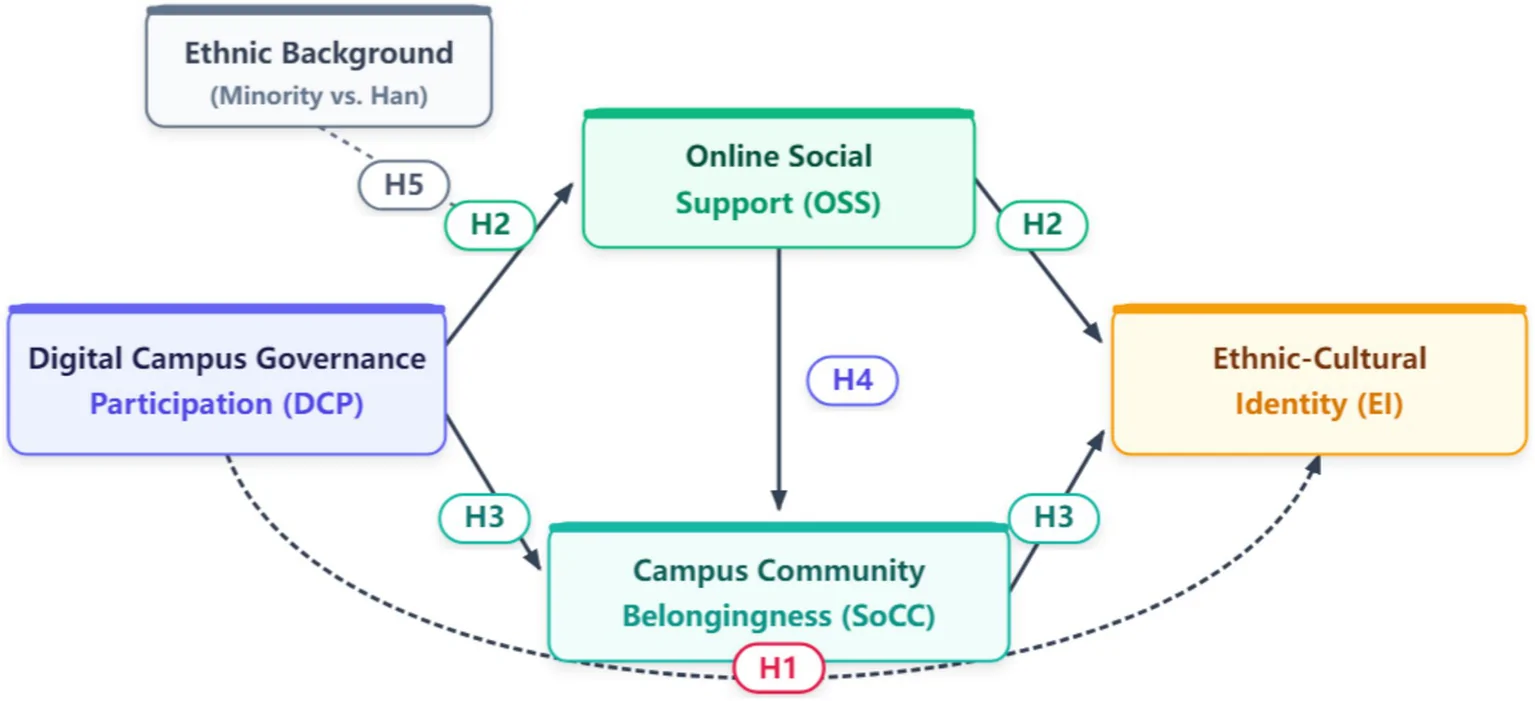
A moderated multiple mediation model of digital campus governance participation and ethnic-cultural identity.

The overall theoretical conceptual model of this study is shown in Figure 2.

## 3 Research design and methods

This study adopts a cross-sectional questionnaire survey design, aiming to examine the statistical associations among digital campus governance participation, online social support, campus sense of community connection, and ethnic-cultural identity.

### 3.1 Sample sampling and data screening

This study focuses its investigation on university student groups in the southwestern region where multi-ethnic students are relatively concentrated. Universities in this region have prominent characteristics of multi-ethnic cultural interaction, and the construction of digital campus service platforms is relatively common, making it suitable for conducting empirical research on the relationship between digital governance participation and ethnic-cultural identity.

The questionnaires were distributed online. The research team collected data through a combination of department stratification and targeted delivery to student groups. Before filling out the questionnaire, all participants read the informed consent instructions, which clearly stated that this study was only for academic analysis, the data would be processed anonymously, participation was completely voluntary, and they could withdraw at any stage.

A total of 950 raw questionnaires were collected in this study. During the data cleaning process, the research team excluded questionnaires with obviously abnormal response times, consecutive identical responses, incorrect answers to attention check questions, and missing key variables. Finally, 890 valid questionnaires were retained, with a validity rate of approximately 93.7%. This sample size can meet the basic requirements of correlation analysis, multiple regression, and Bootstrap mediation association tests.

### 3.2 Measurement tools

All core variables in this study were measured using self-report questionnaires. To ensure the content validity of the translated and adapted scales in the local campus context, the final version of the questionnaire was independently reviewed by researchers in education and psychology before finalization (see Supplementary Material 1 for complete measurement items). The questionnaires uniformly used a 5-point Likert scale, with scores ranging from 1 (“strongly disagree” or “never”) to 5 (“strongly agree” or “always”). Higher scores on each scale indicate higher levels of perception or participation in the corresponding variables.

#### 3.2.1 Digital campus governance participation scale (DCP)

The Digital Campus Governance Participation Scale is mainly used to measure the extent to which students participate in campus digital platforms, online public affairs, and network community interactions. This scale refers to Ribble and Bailey’s (2007) theoretical framework of digital citizenship participation and makes localized adjustments based on digital governance scenes in Chinese universities. The items involve obtaining school notifications, browsing campus forums, participating in online discussions, submitting opinions through the system, and voting on major affairs, with a total of 8 items. In this study, the Cronbach’s *α* of the DCP scale was 0.795, indicating that its internal consistency reached an acceptable level.

#### 3.2.2 Online social support scale (OSS)

The Online Social Support Scale is used to measure the emotional support, informational support, and problem-solving support perceived by students in online environments. This scale refers to the Multidimensional Scale of Perceived Social Support by Zimet et al. (1988) and the Online Social Support Scale by Nick et al. (2018), and limits the context to the Internet and campus digital platforms. The scale contains 5 items, such as “When I encounter difficulties online, I can get help from others.” In this study, the Cronbach’s *α* of the OSS scale was 0.749, and the internal consistency was within an acceptable range.

#### 3.2.3 Sense of community connection scale (SoCC)

The Sense of Community Connection Scale is used to measure students’ sense of membership, influence, integration and fulfillment of needs, and emotional connection to their campus community. This scale mainly refers to the Brief Sense of Community Scale (BSCS) developed by Peterson et al. (2008), with moderate adjustments based on the context of university life. The scale consists of 12 items. While the scale theoretically encompasses four sub-dimensions, the primary objective of this study is to examine the overarching mediating role of community belonging as a holistic psychological resource, rather than comparing the differential effects of specific sub-components. Therefore, in alignment with overarching structural models, the sub-dimensions were aggregated into a single global latent construct for subsequent path analysis. In this study, the Cronbach’s *α* of the SoCC scale was 0.790, indicating that its internal consistency reached an acceptable level.

#### 3.2.4 Ethnic-cultural identity scale (EI)

The Ethnic-Cultural Identity Scale is used to measure students’ subjective identification with their own ethnic background, cultural traditions, and group belonging. This study refers to the Multigroup Ethnic Identity Measure-Revised (MEIM-R) revised by Phinney and Ong (2007), selecting 5 core items based on the local context and highest factor loadings from preliminary adaptations in Chinese adolescent samples, covering two aspects: ethnic identity exploration and commitment. While the original MEIM-R typically demonstrates a high internal consistency (*α* > 0.81), in this study, the Cronbach’s α of the adapted 5-item EI scale was 0.709, reaching the minimum standard usually accepted in social science research.

### 3.3 Data analysis strategy

To meet the strict requirements of Open Science regarding data transparency and reproducibility, this study systematically formulated a detailed dictionary for underlying data cleaning, transformation, and indicator aggregation rules (specific variable renaming, covariate classification anchoring rules, and latent variable score centering logic; see Supplementary Material 2: Research Variable Dictionary and Data Coding Codebook). Based on the standardized underlying data warehouse, subsequent data analysis mainly includes the following core steps:

First, descriptive statistics were used to present the demographic characteristics of the sample, and the risk of common method bias was examined using Harman’s single-factor test. Second, exploratory factor analysis was conducted on the adapted DCP scale to test its structural fit in the sample of this study. Meanwhile, the Cronbach’s *α*, Composite Reliability (CR), and Average Variance Extracted (AVE) of each core scale were calculated to evaluate the internal consistency and convergent validity of the measurement tools. Additionally, confirmatory factor analysis (CFA) fitting indicators and discriminant validity—explicitly utilizing the Fornell–Larcker criterion and the Heterotrait–Monotrait (HTMT) ratio—were comprehensively evaluated to ensure construct independence. Third, Pearson correlation analysis was used to examine the basic associations among DCP, OSS, SoCC, and EI. Fourth, on the basis of controlling demographic variables such as gender, grade, ethnic identity, and university location province, multiple regression analysis and the bias-corrected Bootstrap method (Hayes, 2017) were used to test the multiple mediation association model. The number of Bootstrap resamples was set to 5,000, and whether the 95% confidence interval contained 0 was used to judge whether the indirect association path was significant. Fifth, moderated regression was used to test whether ethnic background moderates the relationship between DCP and OSS.

Regarding variable coding, this study adopted a binary treatment for “ethnic background,” with Han students coded as 0 and ethnic minority students (including Tibetan, Yi, Qiang, Miao, etc.) uniformly coded as 1. This coding strategy was employed to enable a macro-level comparison and ensure the statistical power of the moderated mediation model (e.g., Lai et al., 2024). However, it should be understood as an analytical simplification necessitated by constrained sub-sample sizes, rather than an assumption that different minority groups share homogeneous cultural or adaptation experiences. We recognize that this broad categorization inevitably obscures the nuanced heterogeneity among specific ethnic groups, an analytical limitation that will be explicitly discussed in subsequent sections.

## 4 Data analysis and hypothesis testing

### 4.1 Sample demographic characteristics and common method bias test

The final valid sample size was 890. The sample had good demographic coverage, and the distribution of gender, grade, and ethnic background is shown in Table 1.

**Table 1:** Demographic characteristics of the sample (*N* = 890).

Variable category	Sub-dimension	Frequency (*n*)	Percentage (%)	Cumulative percentage (%)
Gender	Male	403	45.3	45.3
	Female	487	54.7	100.0
Grade	Freshman	323	36.3	36.3
	Sophomore	259	29.1	65.4
	Junior	187	21.0	86.4
	Senior/Graduate	121	13.6	100.0
Ethnic Background	Han	508	57.1	57.1
	Ethnic Minority	382	42.9	100.0

Because the data in this study were mainly from self-report questionnaires, there might be a risk of common method bias. To this end, at the procedural level, this study reduced participants’ evaluation anxiety and social desirability bias by emphasizing the anonymity of the questionnaire and that there were no right or wrong answers (Podsakoff et al., 2003). At the statistical level, Harman’s single-factor test was first used for preliminary evaluation. The unrotated principal component analysis results showed that the variance explained by the first factor was 25.46%, which was far below the common warning standard of 40%. Second, to enhance rigor, this study conducted a full collinearity test using the Variance Inflation Factor (VIF) (Kock, 2015). The test showed that the VIF values of each core latent variable in the model ranged from 1.337 to 2.087, all of which were below the strict threshold of 3.3. In addition, the highest correlation coefficient between core variables was only 0.670 (far below 0.90, Bagozzi et al., 1991). Combining the procedural and multiple statistical diagnostic evidence, the common method bias in this study did not reach a serious level, and the data met the prerequisites for subsequent hypothesis testing.

### 4.2 Structure and measurement quality evaluation of the adapted scale

To ensure the scientific validity and applicability of the measurement tools, this study conducted an exploratory factor analysis on the adapted DCP scale. The results showed that the KMO value was 0.871, and the Bartlett sphericity test reached a significant level (*p* < 0.001), indicating that the data were very suitable for factor analysis.

The principal component analysis results showed that the 8 DCP items formed stable loadings on a single dimension, and the loadings were concentrated between 0.612 and 0.669. This indicates that the scale has very clear structural characteristics and good item convergence in the multi-ethnic university sample of this study. The specific measurement items and exploratory factor loadings are shown in Table 2.

**Table 2:** Exploratory factor loadings of the Digital Campus Governance Participation (DCP) scale.

Measurement item	Construct attribution	Standardized factor loading
DCP_1 Acquiring school notifications	Digital Campus Governance Participation	0.640
DCP_2 Forum browsing frequency	Digital Campus Governance Participation	0.665
DCP_3 Association group data sharing	Digital Campus Governance Participation	0.649
DCP_4 Active posting and leaving messages	Digital Campus Governance Participation	0.632
DCP_5 Daily online discussions	Digital Campus Governance Participation	0.648
DCP_6 Campus sharing on Moments	Digital Campus Governance Participation	0.617
DCP_7 Voting on major affairs	Digital Campus Governance Participation	0.669
DCP_8 Submitting opinions through system	Digital Campus Governance Participation	0.612

The loading cutoff standard was set to 0.40.

Following the exploratory phase, a Confirmatory Factor Analysis (CFA) was conducted to verify the measurement model of the four core latent constructs (DCP, OSS, SoCC, and EI). The model demonstrated an excellent structural fit to the data (*χ*^2^/df = 2.45, CFI = 0.932, TLI = 0.925, RMSEA = 0.040, SRMR = 0.045), confirming the clear structural stability of the scales under a robust sample size (*N* = 890).

In terms of convergent validity, as can be seen from Table 3, except for Online Social Support (OSS, AVE = 0.500), the Average Variance Extracted (AVE) values of Digital Campus Governance Participation (DCP), Sense of Community Connection (SoCC), and Ethnic-Cultural Identity (EI) were all below the common benchmark of 0.500, with the AVE of SoCC being 0.303. However, according to the widely accepted standard proposed by Fornell and Larcker (1981), if the Composite Reliability (CR) of a latent variable is higher than 0.60, the convergent validity of the construct is still considered acceptable in empirical analysis even if its Average Variance Extracted (AVE) falls below 0.50. This pattern of relatively low AVE values is frequently observed in sociological and psychological research when complex, multi-dimensional constructs (such as sense of community and digital citizenship behaviors) are adapted and aggregated into higher-order or single-factor structures for path analysis (Peterson et al., 2008). In this study, the CR values ranged from 0.812 to 0.848. Although these values exceeded the 0.60 threshold, the AVE for SoCC remained notably low (0.303). Therefore, while the internal consistency is adequate, the evidence for convergent validity should be regarded as limited rather than fully satisfactory. Subsequent structural findings involving SoCC should consequently be interpreted with appropriate caution.

**Table 3:** Internal consistency and convergent validity indicators of core measurement tools.

Latent variable	Number of items	Cronbach’s *α*	CR	AVE
Digital Campus Governance Participation (DCP)	8	0.795	0.848	0.412
Online Social Support (OSS)	5	0.749	0.833	0.500
Sense of Community Connection (SoCC)	12	0.790	0.839	0.303
Ethnic-Cultural Identity (EI)	5	0.709	0.812	0.463

### 4.3 Descriptive statistics and correlation analysis

**Figure 3:**
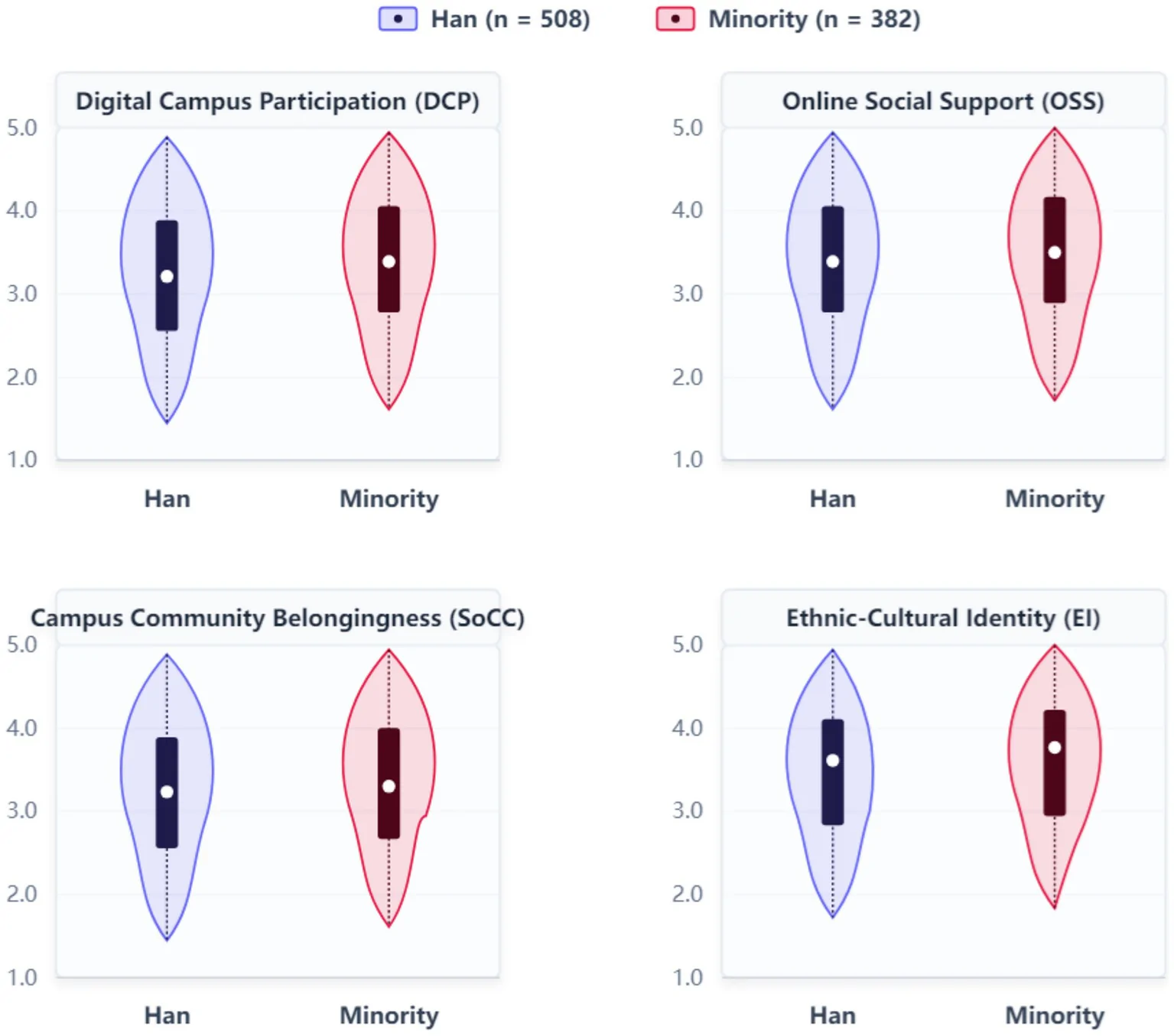
Violin-box plots of core variables grouped by ethnic background.

Before conducting the multiple mediation and path testing, it was necessary to examine the basic distribution characteristics of the four core constructs in the model. Given that this study involves a multi-ethnic university environment and will explore the potential heterogeneous role of ethnic background in the path, the study first plotted smooth kernel density and multi-dimensional box plots for the grouped data of Han students (*n* = 508) and ethnic minority students (*n* = 382) (as shown in Figure 3).

Figure 3 visually displays the grouped distribution characteristics of the four core variables in Han and ethnic minority students. The results show that the scores of each variable present a symmetric distribution close to normal in both groups, and the medians (white dots) and interquartile ranges are highly similar. This confirms that the data have good homogeneity and stability, making them suitable for subsequent path analysis aggregated across the full sample.

On the basis of complete basic distribution states, Table 4 further condenses and presents the means, standard deviations, and Pearson correlation matrix of the four core variables under the full sample. The results indicate that digital campus governance participation (DCP), online social support (OSS), campus sense of community connection (SoCC), and ethnic-cultural identity (EI) all show significant positive statistical associations.

**Table 4:** Descriptive statistics and Pearson correlation matrix of core variables.

Variable	M	SD	1	2	3	4
1. Digital Campus Governance Participation (DCP)	3.21	0.75	(0.642)	0.657	0.845	0.561
2. Online Social Support (OSS)	3.39	0.71	0.508***	(0.707)	0.720	0.577
3. Sense of Community Connection (SoCC)	3.23	0.71	0.670***	0.554***	(0.550)	0.574
4. Ethnic-Cultural Identity (EI)	3.50	0.64	0.422***	0.422***	0.430***	(0.680)

***p < 0.001. Values on the diagonal in parentheses represent the square roots of the AVE. Values below the diagonal are Pearson correlation coefficients, and italicized values above the diagonal are HTMT ratios.

As shown in Table 4, DCP was significantly and positively associated with EI (*r* = 0.422, *p* < 0.001). This basic evidence preliminarily supports research hypothesis H1. Meanwhile, DCP and OSS (*r* = 0.508), DCP and SoCC (*r* = 0.670), and OSS and SoCC (*r* = 0.554) all show moderate to high positive associations. Combined with the preceding VIF results, these correlations did not indicate a serious multicollinearity problem. They also provided a descriptive basis for the subsequent indirect-association analysis.

In addition to examining the correlations among the core variables, discriminant validity was assessed using the Fornell–Larcker criterion and the HTMT ratio. As shown in Table 4, the Fornell–Larcker criterion was not fully satisfied for construct pairs involving SoCC. The HTMT values ranged from 0.561 to 0.845 and remained below the conservative threshold of 0.85, although the DCP–SoCC value was close to this threshold. Thus, the results provide qualified rather than unequivocal support for discriminant validity, and subsequent findings involving SoCC should be interpreted with caution.

### 4.4 Verification of the multiple mediation association model

After controlling for gender, grade, ethnic identity, and university location province, the study further examined the statistical association paths among DCP, OSS, SoCC, and EI. The core regression model parameters are shown in Table 5.

**Table 5:** Stepwise mediation regression analysis (after controlling for covariates).

Regression equation	Indicator variable	Core explanatory variable	Unstandardized coefficient *B*	Standardized coefficient *β*	*t*-value	*p*-value	Δ*R*^2^	Δ*F*
Step A	OSS	DCP	0.483	0.508	17.47	<0.001	0.256	305.3*
Step B	SoCC	OSS	0.287	0.287	10.55	<0.001	0.061	111.4*
	SoCC	DCP	0.495	0.521	19.12	<0.001	0.201	365.4*
Step C	EI	SoCC	0.162	0.178	4.27	<0.001	0.015	18.3*
	EI	OSS	0.207	0.229	6.39	<0.001	0.035	40.8*
	EI	DCP	0.160	0.186	4.63	<0.001	0.018	21.4*

Δ*F* represents the *F*-value change upon the sequential addition of variables in the hierarchical model. *p < 0.001.

The results show that after controlling for demographic variables, there is a significant positive association between DCP and OSS. When both DCP and OSS are included, they both have significant positive relationships with SoCC. When EI is used as the outcome indicator, DCP, OSS, and SoCC all exhibit significant positive statistical associations.

The bias-corrected Bootstrap method was further used for 5,000 resamples to test the multiple indirect association paths among the latent variables (the calculation was constructed based on unstandardized values). The results are shown in Table 6.

**Table 6:** Estimations of multiple indirect associations based on bootstrap.

Indirect Path	Estimate	Standardized estimate	BootSE	95% CI lower limit	95% CI upper limit	Conclusion
DCP → OSS → EI	0.1002	0.117	0.0169	0.0695	0.1357	Significant
DCP → SoCC → EI	0.0799	0.093	0.0197	0.0416	0.1188	Significant
DCP → OSS → SoCC → EI	0.0224	0.026	0.0060	0.0112	0.0349	Significant

Since the 95% confidence intervals of the above three indirect analysis paths did not contain 0, it indicates that online social support (OSS) and campus sense of community connection (SoCC) both constitute significant indirect association bridges between DCP and EI, and the chain path structure formed by the two is also significant. Therefore, research hypotheses H2, H3, and H4 are all supported by empirical data.

**Figure 4:**
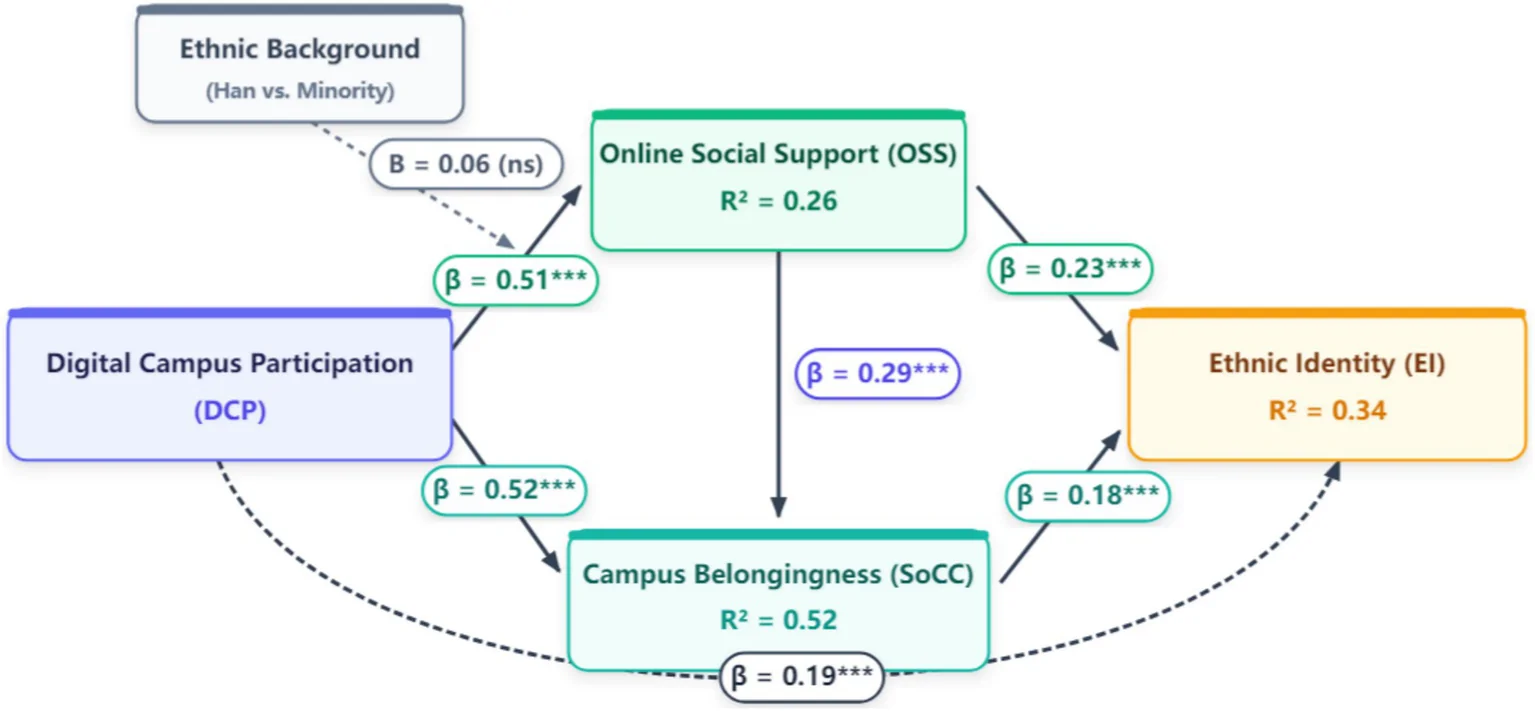
Path test results of the Moderated Chain Mediation Model. *N* = 890. Except for the interaction term (Ethnic Background × DCP) on the path, which is labeled as the unstandardized coefficient *B*, all other coefficients are standardized path coefficients *β*; ns represents not significant, ***p < 0.001 (two-tailed test). The model has controlled for covariates such as gender, grade, ethnic identity, and university location province; the coefficient of determination (*R*^2^) in each endogenous variable box represents the cumulative variance explained by the full model. Data merely reflects cross-sectional covariation and does not constitute causal verification.

To systematically and visually summarize the overall hypothesis testing results of this study, Figure 4 presents the standardized path coefficients and significance after controlling for demographic variables.

From the model paths and coefficient distribution shown in Figure 4, it can be observed that after controlling for covariates, the independent variable digital campus governance participation (DCP) can not only directly and significantly covary with ethnic-cultural identity (EI) (supporting hypothesis H1) but also associate systematically with EI through the three aforementioned indirect pathways (supporting hypotheses H2, H3, and H4). The cumulative explained variance of the full model on ethnic-cultural identity reached 34.0%.

### 4.5 Verification of the moderating association characteristics of ethnic background

To test the heterogeneous path characteristics proposed in Hypothesis H5, this study first conducted a grouped simple slope analysis based on specific groups for Han students and ethnic minority students, comparing the response intensity of the two groups on key paths. The results are shown in Table 7.

**Table 7:** Comparison of unadjusted key path slopes based on ethnic background grouping.

Association path	Group characteristics	Unstandardized slope *B*	Intergroup difference trend
DCP → OSS	Han students (*n* = 508)	0.454	—
DCP → OSS	Ethnic minority students (*n* = 382)	0.517	Difference of 0.063 (not significant)

Grouped regression measurements revealed that the association slope of digital participation (DCP) on online social support (OSS) in the ethnic minority student group was slightly higher than that in the Han student group. This suggests that students under different cultural identity backgrounds have some basic numerical differences in specific paths.

For a more rigorous determination, this study constructed a multiplication interaction term (DCP_c × Minority) between the mean-centered digital participation (DCP_c) and ethnic identity (Minority), and incorporated it into the full-sample regression system to test the statistical significance of the heterogeneous association. The results are shown in Table 8.

**Table 8:** Moderated regression analysis: testing the interaction of DCP and ethnic minority status.

Model parameter	Unstandardized coefficient *B*	Standard error *SE*	*t*-value	*p*-value	Statistical test note
Core Independent Variable (DCP_c)	0.454	0.037	12.166	<0.001	Significant correlation of main variable
Moderating Variable (Minority)	0.005	0.042	0.119	0.905	Non-significant correlation of ethnic identity with dependent variable
Interaction Product (DCP_c × Minority)	0.063	0.055	1.145	0.258	Interaction term did not reach statistical significance

The parameters of the full model show that the association of the core explanatory main variable is highly significant. However, the interactive product term (DCP_c × Minority) representing heterogeneity failed to transcend the statistical significance condition at the 95% confidence level (*B* = 0.063, *SE* = 0.055, *t* = 1.145, *p* = 0.258). Accordingly, the multi-ethnic sample data extracted in this study failed to discover direct statistical evidence supporting that “ethnic background can significantly moderate the association strength between DCP and OSS.” Consequently, Hypothesis H5 was not supported.

**Figure 5:**
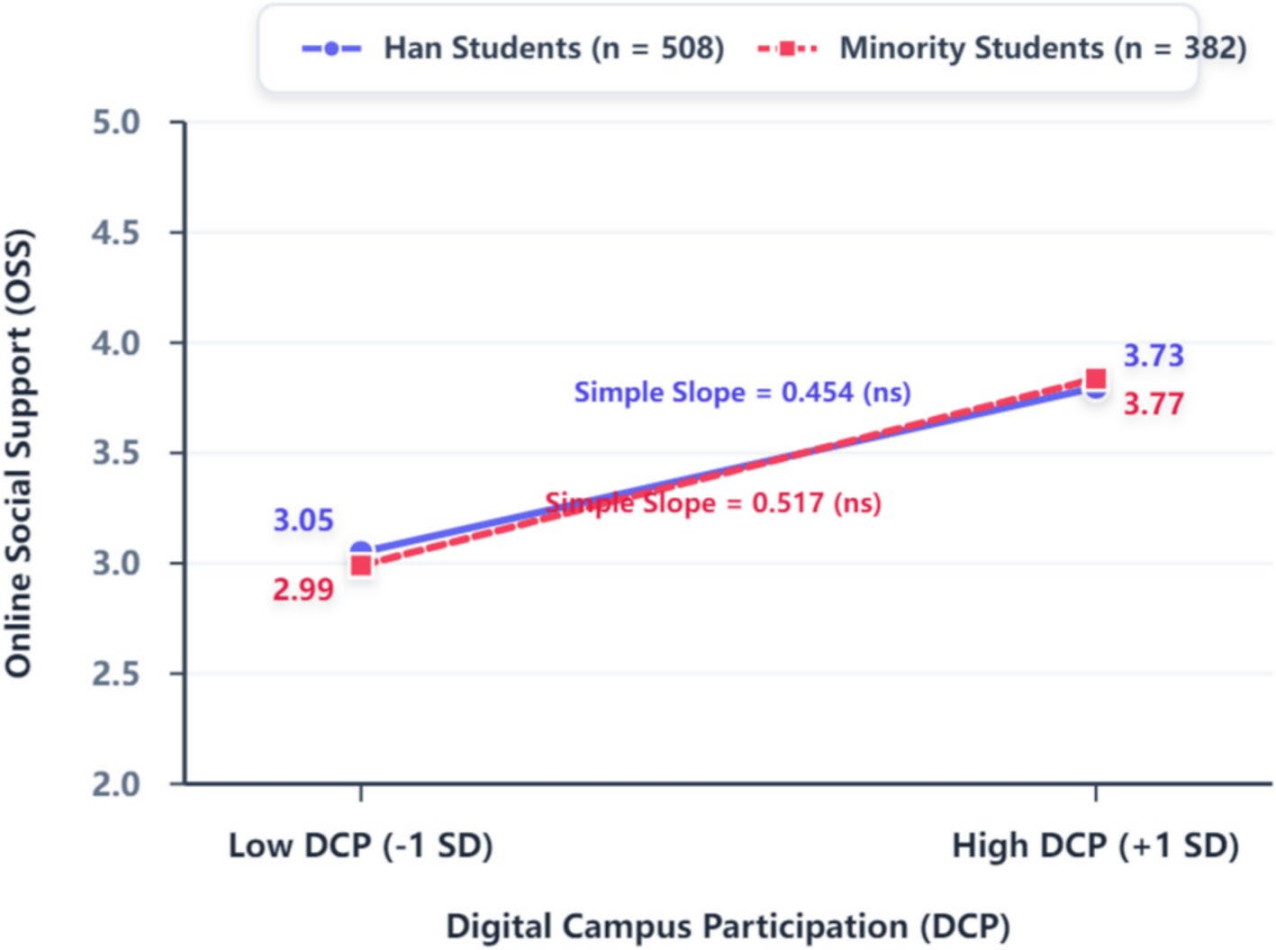
Simple slope diagram of ethnic background in the association between DCP and OSS.

To display this non-significant moderating effect more intuitively, the study plotted a simple slope diagram grouped by ethnic background (its distribution and simulation trajectory at ±1 standard deviation of the variables are shown in Figure 5).

Figure 5 shows a clear parallel trend (Parallel Slopes). Under low digital campus governance participation (Low DCP), the online social support scores of Han students (3.05) and ethnic minority students (2.99) were not different. With the increase of digital participation (High DCP), the online social support of both groups increased at a very similar rate (the Han slope was 0.454, and the ethnic minority slope was 0.517). The two estimated slopes were visually similar, and the interaction term was not statistically significant (*B* = 0.063, *p* = 0.258). Thus, the present analysis did not detect sufficient evidence that the DCP–OSS association differed between the two broadly defined groups. However, the absence of a statistically significant interaction should not be interpreted as proof of equivalence or cross-group invariance, particularly given the binary aggregation of diverse minority groups.

This research conclusion reveals that even if a certain descriptive variation appears at the grouped slope observation level, its strength is not enough to break through individual variation error and transform into significant group heterogeneity features. Therefore, a more stable theoretical landing point should be: under the field conditions and statistical model paradigm established in this study, there is a cross-group similarity stability spectrum between the public participation mechanism of the digital campus dimension and the online social support captured by the group, which is not strictly controlled by the identity boundary between the “Han” and the “ethnic minority framework.”

## 5 Discussion

This study analyzed the relationships among digital campus governance participation, online social support, campus sense of community connection, and ethnic-cultural identity. Based on 890 cross-sectional questionnaire data, the study found that the four core variables are significantly positively correlated, and online social support and campus sense of community connection constitute a significant multiple indirect association path between digital campus governance participation and ethnic-cultural identity. In the heterogeneity test, the interaction term representing ethnic background did not reach the significance level, indicating that this study has not yet found a decisive statistical difference between Han students and ethnic minority students in the relationship strength between DCP and OSS.

### 5.1 Association between digital campus governance participation and ethnic-cultural identity

This study found that digital campus governance participation is significantly positively associated with ethnic-cultural identity. This result provides a new perspective for understanding identity experiences in digital campus environments. The digital campus governance platform is not just a transactional tool; it is also an important space for students to obtain campus information, express demands, participate in public affairs, and perceive community responses.

For multi-ethnic students, digital platforms may lower some barriers to offline expression. Compared with face-to-face expression, online feedback, community discussions, and platform interactions have stronger openness and low-pressure characteristics in some cases. From the perspective of computer-mediated communication (CMC), this “de-individuated” digital space is more likely to induce “hyperpersonal interaction,” allowing individuals to perform highly selective self-presentation and unobstructed identity assertion (Walther, 1996). When students participate in campus life through these platforms, they may more easily feel their position in the campus community. This participation experience is positively associated with ethnic-cultural identity. By providing a complementary, low-friction space for communicative expression, this empirical finding challenges the assumption that digital participation inherently dilutes traditional cultures (Croucher, 2011). Instead, it supports the perspective that digital campus environments can actively correlate with positive identity confirmation by serving as a culturally protective and supportive enclave (Tynes et al., 2012).

### 5.2 Indirect bridging significance of online social support and campus sense of community connection

The core finding of this study is that online social support and campus sense of community connection form a significant multiple indirect association between DCP and EI. This result shows that the association between digital campus governance participation and ethnic-cultural identity is not simply direct but is embedded in a psychological association network composed of perceived support and belonging.

The significant relationship between DCP and OSS indicates that students with higher levels of digital campus governance participation tend to report higher levels of online social support. This is consistent with the daily experience of contemporary college students. Students interact with peers and administrators in campus group chats, online forums, opinion feedback systems, or digital service platforms, which may provide informational help, emotional response, and problem-solving resources. Although these experiences occur online, they are closely related to students’ judgments about whether they are seen, responded to, and supported.

The significant relationship between OSS and SoCC further indicates that online support experiences do not necessarily remain in virtual space. Those who feel more response and help online also tend to report higher campus sense of community connection. This finding empirically supports the “online-to-offline spillover association” of social capital in the digital era (Ellison et al., 2007). In contemporary higher education, the boundary between virtual and physical spaces has become highly permeable. Rather than alienating students from their physical environments, digital interactions serve as a low-friction gateway for resource exchange and relationship maintenance. When students receive timely information and empathetic feedback in digital spaces, these positive online experiences may be cognitively associated with their evaluation of the physical campus, corresponding to a stronger their institutional sense of belonging and community connection. Campus belonging is not generated in the abstract; it often accumulates in repeated problem solving, interaction confirmation, and emotional responses.

The significant result of the chain analysis path DCP → OSS → SoCC → EI further suggests that online support and campus belonging may jointly constitute an important psychological channel connecting digital campus participation and ethnic-cultural identity. For multi-ethnic students, a sense of belonging to the school (SoCC) does not necessarily dilute or assimilate their original ethnic-cultural identity (EI). As conceptualized theoretically earlier based on the Common Ingroup Identity Model and Bicultural Identity Integration Theory, individuals can maintain dual identity orientations (both their cooperative university identity and their distinct ethnic identity) without experiencing psychological conflict, provided the environment is sufficiently inclusive. An inclusive digital governance ecology and a warm online support network essentially lay a “psychological safe haven” for multi-ethnic students. When students experience stronger acceptance and belonging on campus, the potential intergroup threat and defensive anxiety are minimized. This psychological security, in turn, provides minority students with the cognitive flexibility and resilience needed to explore, affirm, and express their unique cultural heritage, thereby realizing the harmonious co-existence of “unity” (school community cohesion) and “diversity” (individual ethnic identity).

It should be noted that while this logically derived mediation sequence aligns with Media Dependency Theory (Ball-Rokeach and DeFleur, 1976), the cross-sectional nature of the data fundamentally captures covariation rather than strict temporal causality. Thus, alternative bidirectional dynamics—such as strong pre-existing cultural identities driving proactive spatial integration—remain a plausible mechanism within this broader psychological network.

### 5.3 Explanation for the non-significant moderating association of ethnic background

This study originally hypothesized that ethnic background might moderate the relationship between digital campus governance participation and online social support. The grouped slope calculation results did show that the slope of DCP and OSS in the ethnic minority student group was slightly higher than that in the Han student group. This descriptive difference is consistent with the judgment that “digital space may provide complementary support channels for ethnic minority students.”

However, the interaction term test incorporating full variable control did not reach a significant level. This result indicates that under the field and analysis paradigm designed in this study, the positive statistical association between digital campus participation and online social support exhibits a similar pattern among Han and ethnic minority students. However, this is not equivalent to asserting that the digital platform experiences of the two groups are completely homogeneous, nor can we easily claim that the university digital governance network has achieved un-differentiated absolute equity and homogeneous inclusion based only on this non-significant result.

This result has two implications. On the one hand, it shows that the association between digital campus governance participation and online social support has high intergroup stability. From the perspective of Socio-Technical Systems Theory, current digital campus governance platforms in multi-ethnic universities (such as general notification pushes, administrative transaction approvals, digital suggestion boxes, etc.) mainly carry highly standardized, universal, and depersonalized public management functions, and their distribution logic and service supply usually do not carry explicit ethnic marks. This homological interface on technical facilities makes the psychological mechanism of transforming digital participation into online support show universality in groups with different cultural backgrounds, which objectively confirms the intergroup equilibrium characteristics of campus digital infrastructure in promoting resource accessibility. On the other hand, non-significant statistical results do not mean that ethnic background is unimportant in more micro-level digital life experiences, nor can we rashly claim that the university digital governance network has achieved absolute equity. Students of different ethnic groups may still have differences in online language encoding habits, safety in cultural psychological expression, reliance on offline co-ethnic social support, and institutional trust in the platform. Critically, while the binary analytical strategy captures macro-level intergroup dynamics, it analytically merges diverse cultural heritages. This broad aggregation inherently averages out linguistic nuances, adaptation stressors, and specific institutional experiences among different minority subgroups. Consequently, rather than indicating an absolute absence of cultural heterogeneity, the non-significant interaction effect might partially reflect an averaging outcome of this macroscopic categorization, which potentially obscures deeper, nuanced variations among specific ethnic groups.

Therefore, the lack of support for H5 is not a failure of the study, but suggests that subsequent research needs to adopt more detailed group classifications and variable measurement methods. For example, we can further distinguish different ethnic minority groups to examine factors such as Mandarin use pressure, frequency of cultural expression, offline co-ethnic support networks, platform trust, and institutional response experience.

### 5.4 Implications for university digital governance practice

The results of this study suggest that the value of university digital governance platforms should not be compressed into transaction handling efficiency. If a platform only carries functions such as notice distribution, leave approval, and administrative assessment, although it improves management convenience, it may not necessarily enhance students’ sense of support and belonging. A digital platform that truly has community significance should simultaneously provide space for expression, response, interaction, and mutual assistance.

For multi-ethnic universities, the construction of digital platforms especially needs to pay attention to inclusive design. Universities must ensure the digital equity of platform interface design under different ethnic backgrounds to avoid digital facilities becoming technical barriers that prevent specific disadvantaged groups from accessing (Tate and Warschauer, 2022). Schools can build digital affinity groups targeting intercultural adaptation or diverse ethnic groups in campus APPs or student online communities, referring to peer group building models (Alicea and Johnson, 2021), to provide a buffer platform for multi-ethnic interaction.

At the same time, universities should also avoid understanding digitalization as a substitute for offline relationships. Research points out that if there is a lack of integration with real-world relationships and excessive reliance on online interaction, it is easy to exacerbate individual social isolation and depression risks (Meshi and Ellithorpe, 2021). Therefore, the ideal governance method is to make online platforms and offline services complement each other, so that students can feel a stable, credible, and sustainable support network in different scenarios.

## 6 Conclusion and outlook

### 6.1 Research conclusion

Taking multi-ethnic university students in the southwestern region as the survey objects, this study extracts the following three core conclusions:

First, there is a significant positive statistical association between digital campus participation and the ethnic-cultural identity of multi-ethnic students. The research results suggest that the higher the level of students’ involvement in campus digital platforms, the higher their reported level of ethnic-cultural identity. This indicates that the experience of digital governance participation is not necessarily a “diluent” of traditional identity, but may instead be accompanied by more positive identity confirmation.

Second, online social support and campus sense of community connection constitute a significant multiple indirect association bridge between DCP and EI. Students’ higher sense of online support in digital governance interactions, together with their stronger campus community connection experience, forms a statistically significant serial indirect association pattern, which collectively bridges the indirect statistical association with their own ethnic-cultural identity.

Third, this study did not observe a statistically significant moderating role of ethnic background in the DCP and OSS path. That is, under the current digital infrastructure background, the covariation pattern between digital participation and perceived social support exhibits consistency across cultural identities. This indirect connection association of digital governance shows certain similarities among student groups of all ethnic groups. The description of this objective law provides a preliminary basis for subsequent how to build an inclusive campus through more refined means.

### 6.2 Research limitations

This study still has several limitations.

First, the study adopts a cross-sectional questionnaire design, and all variables were measured at the same time point, so the temporal order of variables cannot be confirmed. Although the mediation model fits theoretical expectations and obtained statistical support through the Bootstrap method, these paths can only be interpreted as indirect associations, not as causal chains. For instance, it is equally plausible that students who already possess a strong ethnic-cultural identity naturally take the initiative to engage with campus communities, leading them to utilize digital governance platforms more actively. Such bidirectional dynamics or reverse causal pathways naturally exist in real-world contexts and cannot be definitively ruled out by the current research design.

Second, the data mainly come from student self-report questionnaires. Although the Harman single-factor test and the full collinearity VIF test comprehensively show that the common method bias did not reach a serious level, self-reports may still be affected by social desirability, immediate emotions, and response contexts. Especially on relatively sensitive topics such as ethnic-cultural identity, some students may tend to give answers that are more in line with social expectations.

Third, the measurement structure of scales such as Sense of Community Connection (SoCC) in this study faces tension between conceptual multidimensionality and single-dimensional aggregation. The SoCC scale used in this study is derived from the Brief Sense of Community Scale (BSCS) developed by Peterson et al. (2008). Theoretically, this scale spans four core dimensions: membership, influence, integration and fulfillment of needs, and shared emotional connection. When this study aggregates it as a high-order single-dimensional variable to match the macro-level structural model, it successfully captures the global psychological resource of community belonging. However, this approach inevitably collapses unique item-level variances into a single factor, which computationally bounded the Average Variance Extracted (AVE), despite the adequate composite reliability and model fit. Future research, if the sample size permits, can try to use a Bifactor Model or Second-Order Confirmatory Factor Analysis (CFA) to effectively isolate and control the variation of non-target dimensions, so as to further improve the purity of key construct measurements.

Fourth, in terms of conceptual operationalization of ethnic background, this study adopted a binary design of “Han/ethnic minority.” Although this treatment meets the strict requirements of statistical models for simplified models and interaction term test power, it inevitably obscures the heterogeneous ecology within the “diverse ethnic groups” to a certain extent. Ethnic minority students in southwestern Chinese universities include multiple groups such as Tibetan, Yi, Hui, and Qiang. Different groups exhibit distinct behavioral characteristics in language habits, religious beliefs, cultural distances of native communities, and transitional adaptation periods to the mainstream campus culture. Merging diverse ethnic identities into a single “ethnic minority” category may mask the unique micro-psychological patterns of specific ethnic groups in digital campus governance participation (DCP) and online social support seeking. Future research should expand the sample size of specific ethnic minorities through targeted over-sampling, so as to have the prerequisite conditions for conducting multigroup path analysis (Multigroup CFA/SEM).

Fifth, although the study controlled for variables such as gender, grade, ethnic identity, and university location province, there are still some potential confounding variables not included in the model. At the same time, the description of students’ “intersectionality” is still relatively flat. The latest intergroup mental health and adaptation research repeatedly emphasizes that ethnic/racial backgrounds can present the true psychological experience of youth in marginalized positions only when they are placed in a framework where gender, sexual orientation, and specific status intersect (Simon-Kumar et al., 2025). These unobserved confounding variables may be associated with digital participation, online support, and ethnic-cultural identity, thereby constituting certain theoretical limitations to the explanatory power of the existing regression framework.

### 6.3 Future research directions

Subsequent research can continue to advance from several directions.

A more important direction is to adopt a longitudinal tracking design, repeatedly measuring digital campus governance participation, online social support, campus sense of community connection, and ethnic-cultural identity at different stages of students’ enrollment, adaptation, stability, and graduation. Through longitudinal data and cross-lagged models, future research can more clearly examine the temporal changes between variables.

Future research can also combine interviews, focus groups, or digital ethnography methods to deeply understand students’ real experiences in specific platforms. Quantitative models can reveal the overall association between variables, but it is difficult to fully present how students form a specific sense of support and belonging in a single online help-seeking, a cultural discussion, or a piece of campus affairs feedback. Qualitative data can make up for this deficiency.

In addition, subsequent research can further refine ethnic background variables, not only distinguishing Han and ethnic minorities, but also examining differences among different ethnic minority groups when the sample size permits. At the same time, variables such as Mandarin use pressure, safety in cultural expression, offline co-ethnic networks, platform trust, and institutional response quality can also be included, so as to understand in more detail how digital campus governance intertwines with multi-ethnic students’ daily interactions, community belonging, and identity.

## Data Availability

The original contributions presented in the study are included in the article/Supplementary material, further inquiries can be directed to the corresponding author.
