# Supplementary material for: Digital campus governance and cultural identity of multi-ethnic students: a multiple mediation analysis of online social support and sense of community connection
Source: Front Psychol. 2026 Aug 20;17:1918341. doi: 10.3389/fpsyg.2026.1918341 (PMC13537989; doi:10.3389/fpsyg.2026.1918341)
Supplement: Supplementary file 1 [file Data_Sheet_1.ZIP › Supplementary_Material_2_Research Variable Dictionary and Data Codebook.docx]

Supplementary Material 2: Research Variable Dictionary and Data Codebook

This codebook details the variable naming rules, item anchors, and assignment systems of all core latent variables and covariates in the underlying dataset of this study (*N* = 890) to facilitate data review and model replication.

**Table I. Demographic Covariates and Categorical Coding**

| Variable | Role in Analysis | Questionnaire Item Description | Coding Scheme |
| --- | --- | --- | --- |
| Gender | Covariate | What is your gender? | Categorical: 1 = Male; 2 = Female |
| Grade | Covariate | What is your current grade? | Ordinal: 1 = Freshman; 2 = Sophomore; 3 = Junior; 4 = Senior; 5 = Postgraduate and above |
| Ethnicity | Raw Demographics | What is your ethnicity? | Categorical: 1 = Han; 2 = Tibetan; 3 = Yi; 4 = Qiang; 5 = Miao; 6 = Other ethnic groups |
| Minority | Moderator / Covariate | (Dummy variable converted from Ethnicity) | Dummy: 0 = Han student (Han, Ethnicity=1); 1 = Ethnic minority student (Minority, Ethnicity options 2-6) |
| Province | Covariate | Your university location province  ? | Categorical: 1 = Sichuan Province; 2 = Yunnan Province; 3 = Guizhou Province. (Note: Selecting other provinces will directly terminate the questionnaire, and it is used as a control factor in the regression to absorb variance) |

**Table II. Core Latent Constructs and Continuous Scoring System**

Note: All psychological and behavioral constructs below use a 5-point Likert scale. The scoring standard is uniformly: 1 = “Strongly Disagree (or Never)”, 2 = “Disagree (or Rarely)”, 3 = “Neutral (or Sometimes)”, 4 = “Agree (or Often)”, 5 = “Strongly Agree (or Always)”. Before entering correlation and regression analysis, high-order latent variable scores are generated by aggregating the mean values of the corresponding items.

| Item Set | Construct | Items Included in Dataset | Mean Aggregation Logic |
| --- | --- | --- | --- |
| DCP_1 to DCP_8 | Digital Campus Governance Participation (Digital Community Participation) | 8 items in total (measuring: notification acquisition, forum browsing, data sharing, online posting, discussion, campus sharing, online voting, system submission). Note: Question 9 in the original questionnaire is an “attention check question,” which is only used for the preliminary screening of invalid samples and does not participate in the final variable score calculation. | DCP = Mean(DCP_1 to DCP_8) Continuous numeric value (1.00 - 5.00) |
| DCP_c | Mean-Centered DCP | (Manually generated high-order variable used to construct the mean-centered interaction main variable to avoid the risk of collinearity inflation) | DCP_c = DCP - Mean(DCP full sample) Continuous numeric value |
| OSS_1 to OSS_5 | Online Social Support | 5 items in total (measuring: online difficulty help, listening/companionship, easy friend-making, emotional comfort, timely response) | OSS = Mean(OSS_1 to OSS_5) Continuous numeric value (1.00 - 5.00) |
| SoCC_1 to SoCC_12 | Sense of Community Connection | 12 items in total (measuring community evaluation in dimensions such as sense of school membership, bi-directional influence, peer need collaboration, shared emotional connection) | SoCC = Mean(SoCC_1 to SoCC_12) Continuous numeric value (1.00 - 5.00) |
| EI_1 to EI_5 | Ethnic-Cultural Identity | 5 items in total (measuring: ethnic cultural background exploration, custom/activity heritage, clear group cognition, collective pride, strong sense of internal connection) | EI = Mean(EI_1 to EI_5) Continuous numeric value (1.00 - 5.00) |
| DCP_c × Minority | (Interaction Term) | Multivariate statistical product variable used to test Hypothesis H5, constructed based on the multiplication of the centered main variable and the ethnic minority dummy variable group. | Interaction = DCP_c * Minority Continuous numeric value |
