# Supplementary material for: Digital campus governance and cultural identity of multi-ethnic students: a multiple mediation analysis of online social support and sense of community connection
Source: Front Psychol. 2026 Aug 20;17:1918341. doi: 10.3389/fpsyg.2026.1918341 (PMC13537989; doi:10.3389/fpsyg.2026.1918341)
Supplement: Supplementary file 1 [file Data_Sheet_1.ZIP › Supplementary_Material_1_Survey Questionnaire.docx]

Digital Campus Governance and Cultural Identity of Multi-ethnic Students

Dear Student:

Hello!

We are a research team from Sichuan Minzu University, and we sincerely invite you to participate in an academic study on college students’ digital life and psychological experiences.

This study aims to understand how contemporary college students from diverse cultural backgrounds participate in campus life through digital platforms, and how these online interaction experiences shape your campus sense of belonging and your own cultural/ethnic identity. Your answers are crucial to our understanding of how to build a more inclusive digital campus community that can better meet the psychological expectations of students of all ethnic groups.

【Important Notes】

Complete Anonymity: This questionnaire is conducted in a completely anonymous manner. Your personal information (such as ethnicity, gender, etc.) is only used for overall statistical analysis. We will strictly observe academic ethical norms, keep your personal identity and answers absolutely confidential, and all data will be presented only in aggregate form and used for academic exchange, so please feel free to answer.

Voluntary Participation: This survey is completely voluntary. You can choose to suspend or exit the answering process at any time. If you complete the questionnaire and click “Submit”, we will assume that you agree to participate in this study.

Estimated Duration: Completing this questionnaire takes about 8-10 minutes.

Honest Answering: There are no “right” or “wrong” answers to the questions in the questionnaire. We care about your true feelings and experiences. Please simply make your choice based on your first reaction.

Thank you very much for your valuable time and strong support!

Part One: Basic Information

1.What is your current grade?

Freshman

Sophomore

Junior

Senior

Postgraduate and above

2.What is your gender?

Male

Female

3.What is your ethnicity?

Han

Tibetan

Yi

Qiang

Miao

Other ethnic groups

4.In which province is your current university located?

Sichuan Province

Yunnan Province

Guizhou Province

Other provinces (If this option is selected, the questionnaire ends)

**Part Two: Digital Community Participation**

(Note: “Digital community” refers to various platforms and communities related to your campus life that you participate in through online methods such as mobile Apps, websites, WeChat groups, and QQ groups.)

Please evaluate how frequently you perform the following behaviors based on your actual situation in the most recent semester.

(1=Never, 2=Rarely, 3=Sometimes, 4=Often, 5=Always)

| Item | Never | Rarely | Sometimes | Often | Always |
| --- | --- | --- | --- | --- | --- |
| 1. Obtain school notifications and announcements through the official campus website, official App, or WeChat official account. | ○ | ○ | ○ | ○ | ○ |
| 2. Browse posts or information on the campus BBS/forum/Tieba. | ○ | ○ | ○ | ○ | ○ |
| 3. Obtain study materials or activity information through various online channels (such as course groups, club groups). | ○ | ○ | ○ | ○ | ○ |
| 4. Post or leave comments on campus-related social platforms (such as BBS, Tieba, campus topics on Douyin/Bilibili). | ○ | ○ | ○ | ○ | ○ |
| 5. Conduct daily communication and discussion with classmates in campus communities such as class groups, club groups, and fellow-townsman groups. | ○ | ○ | ○ | ○ | ○ |
| 6. Share content related to campus life through online platforms (such as QQ Zone, Moments). | ○ | ○ | ○ | ○ | ○ |
| 7. Participate in voting for campus affairs (such as the “My Favorite Teacher” selection) through online channels. | ○ | ○ | ○ | ○ | ○ |
| 8. Propose opinions or suggestions regarding school management or services through online channels (such as the President’s mailbox, logistics repair platform). | ○ | ○ | ○ | ○ | ○ |
| 9. For questionnaire quality assurance, please select “Often” for this question. | ○ | ○ | ○ | ○ | ○ |

**Part Three: Personal Feelings and Experiences**

Please evaluate the extent to which you agree with the following statements based on your true feelings.

(1=Strongly Disagree, 2=Disagree, 3=Neutral, 4=Agree, 5=Strongly Agree)

**A. Online Interaction Perceptions**

| Item | Strongly Disagree | Disagree | Neutral | Agree | Strongly Agree |
| --- | --- | --- | --- | --- | --- |
| 1. When I encounter difficulties in life or study, I can find useful advice in campus-related online communities. | ○ | ○ | ○ | ○ | ○ |
| 2. I feel that many classmates truly care about me in online communities (such as fellow-townsman groups, club groups). | ○ | ○ | ○ | ○ | ○ |
| 3. I can easily find friends I click with through online means. | ○ | ○ | ○ | ○ | ○ |
| 4. My friends in campus online communities provide me with emotional support (such as comfort, encouragement). | ○ | ○ | ○ | ○ | ○ |
| 5. When I ask questions on school-related websites or Apps, I usually receive a timely response. | ○ | ○ | ○ | ○ | ○ |

**B. Personal Identity**

| Item | Strongly Disagree | Disagree | Neutral | Agree | Strongly Agree |
| --- | --- | --- | --- | --- | --- |
| 1. I spend a lot of time trying to learn more about the history and culture of my ethnic group. | ○ | ○ | ○ | ○ | ○ |
| 2. I often participate in cultural activities (such as festivals, rituals) related to my ethnic group. | ○ | ○ | ○ | ○ | ○ |
| 3. I have a clear understanding of my sense of belonging to my ethnic group. | ○ | ○ | ○ | ○ | ○ |
| 4. I am happy to be a member of this ethnic group. | ○ | ○ | ○ | ○ | ○ |
| 5. I feel a strong connection with other members of my ethnic group. | ○ | ○ | ○ | ○ | ○ |

**C. Campus Community Perceptions**

| Item | Strongly Disagree | Disagree | Neutral | Agree | Strongly Agree |
| --- | --- | --- | --- | --- | --- |
| 1. I feel that I am a member of our school’s big family. | ○ | ○ | ○ | ○ | ○ |
| 2. I know most of the people in our school. | ○ | ○ | ○ | ○ | ○ |
| 3. Most students in the school know me. | ○ | ○ | ○ | ○ | ○ |
| 4. My opinions and views are valued at school. | ○ | ○ | ○ | ○ | ○ |
| 5. I have an influence on the future development of our school. | ○ | ○ | ○ | ○ | ○ |
| 6. I can get what I need at school. | ○ | ○ | ○ | ○ | ○ |
| 7. My classmates and I have similar values. | ○ | ○ | ○ | ○ | ○ |
| 8. I feel that my classmates and I can cooperate well to accomplish things together. | ○ | ○ | ○ | ○ | ○ |
| 9. I expect to maintain close contact with my alma mater (or school teachers and classmates) after graduation. | ○ | ○ | ○ | ○ | ○ |
| 10. I think my school is a great place to live and study. | ○ | ○ | ○ | ○ | ○ |
| 11. Compared to other schools, I prefer our school. | ○ | ○ | ○ | ○ | ○ |
| 12. Daily interaction with classmates is very important to me. | ○ | ○ | ○ | ○ | ○ |

Thank you very much once again for taking time out of your busy schedule to participate in this survey! Your support is of great significance to our research work.

Wish you academic progress and all the best!
